# Supplementary material for: Serum albumin and gamma gap levels, and combined effect for risk of mortality in a Japanese population from the J-MICC study
Source: PLoS One. 2025 Nov 17;20(11):e0336926. doi: 10.1371/journal.pone.0336926 (PMC12622803; doi:10.1371/journal.pone.0336926)
Supplement: S1 File — (DOCX) [file pone.0336926.s001.docx]

J-MICC study has been conducted with the contribution of many people listed as below. We would like to express our gratitude to them.

Chiba Cancer Center: Nobuaki Michihata, Yohko Nakamura, Haruo Mikami, Hiroki Nagase, Kimiko Takayama

Kanagawa Cancer Center: Hiroto Narimatsu, Sho Nakamura, Kaname Watanabe, Kayoko Katayama, Yoshinobu Saito, Ryoko Kawakami, Chei Choy Lye, Masumi Okamoto, Naoko Shinmura, Koshi Takahashi

Shizuoka Prefectual University: Kiyonori Kuriki

Nagoya City University: Sadao Suzuki, Takeshi Nishiyama, Takahiro Otani, Hiroko Nakagawa, Shinkan Tokudome, Akihiro Hosono, Yuya Tamai, Miki Watanabe, Makoto Sunayama

Aichi Cancer Center: Keitaro Matsuo, Hidemi Ito, Isao Oze, Yuriko Koyanagi, Yuji Iwashita, Yumiko Kasugai, Masanori Kawaura, Kazuo Tajima, Wakai Kenji, Kaoru Hirose, Akio Hiraki, Hideo Tanaka, Takeshi Suzuki, Takakazu Kawase, Satoyo Hosono, Yukari Taniyama

Nagoya University: Kenji Wakai, Takashi Tamura, Mako Nagayoshi, Takashi Matsunaga, Rieko Okada, Yoko Kubo, Yoko Mitsuda, Yasufumi Kato, Hidemi Hattori, Masahiro Nakatochi, Yoshiko Ishida, Satoshi Osafune, Yuka Kadomatsu, Sayo Kawai, Etsuko Kimura, Sayaka Kuriki, Tae Sasakabe, Yuka Sugimoto, Shino Suma, Toshio Seiki, Sahoko Takagi, Kenji Takeuchi, Akiko Tamakoshi, Yudai Tamada, Mineko Tsukamoto, TowaTai（Yin Guang）, Kotaro Tomita, Mariko Naito, Hiroko Nakagawa, Kazuko Nishio, Yuta Hattori, Nobuyuki Hamajima, Takahiro Higashibata, Asahi Hishida, Nana Fukuda, Kaori Masui, Kenta Maruyama, Keiko Mizutani, Emi Morita

Shiga Medical University/Tsuruga Nursing University: Kuniyoshi Kita, Katsuyuki Miura, Yasuyuki Nakamura, Naotaka Takashima, Kenji Matsui, Aya Kadota, Tanvir Chowdhry Turin, Naoko Miyagawa, Hiroshi Ueshima, Fusako Katsurada, Masae Torii, Etsuko Maekawa

Kyoto Prefectual University of Medicine: Teruhide Koyama, Etsuko Ozaki, Naoyuki Takashima, Daisuke Matsui, Isao Watanabe, Reo Nagamitsu, Yoshiyuki Watanabe, Ritei Uehara, Nagato Kuriyama, Satomi Tomida, Yukiko Nukaya, Mizuho Wada, Komei Iwai, Chie Omichi, Rika Tanaka, Fumitaro Miyatani, Mao Hirota, Aya Yoshikawa, Kumiko Hara, Satoko Mitani

Tokushima University: Kokichi Arisawa, Sakurako Kamano, Hirokazu Uemura, Miwa Yamaguchi, Mineyoshi Hiyoshi, Mariko Nakamoto, Masashi Ishizu, Takeshi Watanabe, Noriko Tsuruta, Natsuko Yamamoto, Momoko Yamaguchi, Manami Inohara, Rie Matsumura, Yayoi Asano, Hidenobu Takami, Tirani Bahari, Yuki Iwasaki, Tien Van Nguyen

Kyushu University: Jun Hayashi, Norihiro Furusho, Hiroaki Ikezaki, Suminori Kono, Keizo Ohnaka, Makiko Umemoto, Ryoko Nakashima

Saga University: Keitaro Tanaka, Megumi Hara, Yuichiro Nishida, Chisato Shimanoe, Takuma Furukawa, Hinako Nanri, Yasuki Higaki, Koichi Shinchi

Kagoshima University: Chihaya Koriyama, Shiro Tanoue, Toshiro Takezaki, Hideshi Niimura, Rie Ibusuki, Kazuyo Kuwabara, Noriko Nakahata, Masaya Tatebo, Keiichi Shimatani, Ippei Shimoshikiryo, Daisaku Nishimoto, Kenichi Shibuya, Rika Matsuyama, Chiharu Takada, Yoshifumi Hidaka, Motahareh Kheradmand, Eva Mariane Mantjoro, Tara Sefanya Kairupan, Yora Nindita, Athira Dhruva, Saekhol Bakri, Inria Astari Zahra

Aichi Medical University: Asahi Hishida

Research Supporting Group: Kenji Wakai, Hidetaka Eguchi, Naomi Imaeda, Chiho Goto, Yukihide Tomozawa, Tomohiro Shinozaki, Shuji Hashimoto, Takashi Takahashi, Akihiro Sekine, Kei Nakachi, Kazue Imai, Michiaki Kubo
