# Supplementary material for: Serum albumin and gamma gap levels, and combined effect for risk of mortality in a Japanese population from the J-MICC study
Source: PLoS One. 2025 Nov 17;20(11):e0336926. doi: 10.1371/journal.pone.0336926 (PMC12622803; doi:10.1371/journal.pone.0336926)
Supplement: Attachment — Submitted filename: Recommendation and Comments for PONE-D-24-16646.docx [file pone.0336926.s002.docx]

The following list of issues needs to be addressed:

1. Throughout the manuscript some statements lack of reference. You should add more updated reference.
2. Discuss further details about the function of albumin how it maintains the oncotic pressure as well as how it contributes to transport, solubilization and stabilization.
3. The manuscript would be more understandable after the inclusion of a flow diagram of study design that outlines participant recruitment, inclusion and exclusion criteria, and the ultimate sample size.
4. The measurement technique and kits used for the quantification of total serum protein and albumin are not described. Clarify the information in the materials and methods section.
5. What criteria did you use to separate the age groups of 35–49, 50–59, and 60–69 years? In the statistical part, explain it.
6. Mean or median of serum total protein, albumin, and gamma gap level for male and female is necessary for a better understanding.
7. What type of information you are providing or recommending to the general population through this study.
8. Please briefly discuss the physiological and clinical significance of low albumin and high levels of gamma gap level in respect to the diseases.
9. There have repetitions of some information and typos error. You should revise the manuscript properly.
10. Show the relationship among serum total protein, albumin, and gamma gap levels, if there have.
