# Supplementary material for: Serum albumin and gamma gap levels, and combined effect for risk of mortality in a Japanese population from the J-MICC study
Source: PLoS One. 2025 Nov 17;20(11):e0336926. doi: 10.1371/journal.pone.0336926 (PMC12622803; doi:10.1371/journal.pone.0336926)
Supplement: Attachment — Submitted filename: Reviewer2.docx [file pone.0336926.s004.docx]

Manuscript Number: PONE-D-24-16646

Serum albumin and gamma gap levels, and combined effect for risk of mortality in a Japanese population from the J-MICC study

Dear Reviewer 2:

On behalf of my co-authors, we would like to express our great appreciation to the reviewers. We have carefully taken the reviewers’ comments into consideration in the preparation of our revision. All changes to the reviewer 1 and reviewer 2 are combined and are highlighted in yellow maker in the manuscript. We have included point-to-point response to the reviewer’ comments as follows.

1. Throughout the manuscript some statements lack of reference. You should add more updated reference.

We added and updated references. We also deleted the sentences without reference in **Discussion**.

[update]

1.	Henry's Clinical Diagnosis and Management by Laboratory Methods. 24 ed. Philadelphia, PA: Elsevier; 2021.

2.	Serum Albumin: Structure, Functions and Health Impact (Protein Biochemistry, Synthesis, Structure and Cellular Functions). New York: Nova Science Pub Inc; 2012.

[add]

4. Moramarco S, Morciano L, Morucci L, Messinese M, Gualtieri P, Carestia M. Epidemiology of Hypoalbuminemia in Hospitalized Patients: A Clinical Matter or an Emerging Public Health Problem? Nutrients. 2020 Nov 27;12(12):3656. doi: 10.3390/nu12123656. PubMed: PMC7760225.

5. Oster HS, Dolev Y, Kehat O, Weis-Meilik A, Mittelman M. Serum Hypoalbuminemia Is a Long-Term Prognostic Marker in Medical Hospitalized Patients, Irrespective of the Underlying Disease. J Clin Med. 2022;11(5):1207. doi: 10.3390/jcm11051207. PubMed: PMC8911288.

2. Discuss further details about the function of albumin how it maintains the oncotic pressure as well as how it contributes to transport, solubilization and stabilization.

We added further details about the function of albumin in **Introduction**.

- **Introduction**, line 59-77; “Albumin is a negatively charged protein with a high concentration and relatively large molecular weight, accounting for approximately 75–80% of plasma colloid osmotic pressure [2]. The negative charge of the albumin molecule also attracts cations and water, contributing to water retention within capillaries and playing a crucial role in stabilizing blood volume [2]. Furthermore, albumin molecules possess various ligand-binding sites, enabling them to bind and transport substances such as fatty acids, hormones, bilirubin, and calcium [2]. Approximately 40% of the calcium in whole blood exists in a bound state with albumin, which buffers and stabilizes blood calcium levels [1]. Highly hydrophobic molecules also become soluble in blood by binding to albumin, allowing them to be transported to target tissues [2]. Furthermore, albumin binds to drugs and toxins, suppressing the concentration of free active forms and mitigating rapid onset of action or toxicity [2]. Physiologically, low albumin levels impair the maintenance of adequate plasma colloidal osmolarity. This makes it easier for fluid to leak from blood vessels into tissues, potentially causing edema, pleural effusion, and ascites. Furthermore, as albumin transports various substances, its reduction serves as an indicator of conditions such as malnutrition, liver dysfunction, and chronic inflammation. Clinically, hypoalbuminemia is a poor prognostic indicator, with numerous studies showing a strong association with increased mortality in hospitalized patients and those with chronic diseases [4, 5].”

3. The manuscript would be more understandable after the inclusion of a flow diagram of study design that outlines participant recruitment, inclusion and exclusion criteria, and the ultimate sample size.

We added a flow diagram in Fig 1.

- **Figure Legend**, page 45; Fig 1. Flow diagram for selecting eligible participants from 13 cohorts.

4. The measurement technique and kits used for the quantification of total serum protein and albumin are not described. Clarify the information in the materials and methods section.

We added the measurement technique and kits used for the quantification of total serum protein and albumin in **Methods/Clinical characteristics**.

- **Methods/Clinical characteristics**, line 162-165; “Examination of serum total protein levels was carried out using the Biuret method. Serum albumin levels were examined using bromocresol green (BCG) test or modified bromocresol purple (BCP) test.”

5. What criteria did you use to separate the age groups of 35-49, 50-59, and 60-69 years? In the statistical part, explain it.

We added the criteria of separated age group in **Methods**/**Statistical analyses**.

- **Methods**/**Statistical analyses**, line 183-184; “Age was divided into three groups (35–49, 50–59, and 60–69 years) according to similar number of participants.”

6. Mean or median of serum total protein, albumin, and gamma gap level for male and female is necessary for a better understanding.

We added the mean and standard deviation of serum total protein, albumin, and gamma gap level criteria of separated age group in **Results** and Table 1.

- **Results**, line 250-252; “The mean levels of serum total protein, albumin and gamma gap in men and women were 7.22 and 7.30; 4.45 and 4.43; and 2.77 and 2.87, respectively.”

7. What type of information you are providing or recommending to the general population through this study.

We added the information providing or recommending to the general population through this study in **Conclusion**. We also added the description on its related results in **Abstract** and **Discussion**.

- **Conclusion**, line 417-421; “The population with low albumin or high gamma gap even without specific diseases may be required careful follow-up in health checkup. Especially, particular attention may be given to people with low albumin and high gamma gap for RSD without pneumonia, and low albumin and low gamma gap for pneumonia.”
- **Abstract**, line 18-22; “Increased HRs of low albumin and high gamma gap levels were respectively observed for deaths from all-causes, cancer, cardiovascular diseases, respiratory system diseases without pneumonia, and other-causes; and the HR was the highest on respiratory system diseases without pneumonia (HR = 7.31, 4.15-12.9). Low albumin and low gamma gap levels were strongly associated for pneumonia death (HR = 12.4, 3.98-38.5).”
- **Discussion**, line 371-373; “The HRs for any deaths, except pneumonia, with low albumin and high gamma gap levels was higher than those with low albumin and medium gamma gap levels, and the HR was the highest for RSD without pneumonia.”
- **Discussion**, line 376-379; “On the other hand, low albumin and low gamma gap levels were strongly associated for pneumonia death, and significant interaction was also observed between low albumin and low gamma gap levels for pneumonia death.”

8. Please briefly discuss the physiological and clinical significance of low albumin and high levels of gamma gap level in respect to the diseases.

We added the description on the physiological and clinical significance of low albumin and high levels of gamma gap level in respect to the diseases, although the present results did not suggest the risk of specific diseases, except pneumonia.

- **Introduction**, line 51-58; “Therefore, in addition to insufficient nutritional intake, serum albumin levels decline due to impaired absorption of amino acids because of a breakdown in the gastrointestinal mucosal epithelium, decreased synthesis capacity in the liver from cirrhosis, protein leakage from the gastrointestinal tract and kidneys due to protein-leaking gastroenteropathy and nephrotic syndrome, and hyper-catabolism from severe infections and chronic inflammatory diseases. Among them, production disorder has a great influence on a serum albumin decrease, which depends on the ability of the liver to synthesize [3].
- **Introduction**, line 91-97; “Physiologically, a high gamma gap suggests increased immunoglobulin levels. This indicates the possibility of ongoing chronic inflammation within the body or abnormal plasma cell proliferation. Clinically, it can serve as a differential diagnostic indicator for conditions such as multiple myeloma, macroglobulinemia, and primary amyloidosis. It is also used as a screening indicator for plasma cell disorders, and when a marked elevated gamma gap is observed, further investigation such as protein electrophoresis is recommended.”
- **Discussion**, line 352-354; “Various factors which were associated with gamma gap levels in cross-sectional observation are concordant with those for latent inflammation, cancer, and autoimmune diseases, in which the gamma gap is used as a clinical screening index [7-10].”
- **Discussion**, line 363-366; “This study suggests that high serum gamma gap levels are an indicator of a high risk for mortality due to various diseases in a Japanese population, which may be involved in chronic inflammation across the lifespan [29].”

9. There have repetitions of some information and typos error. You should revise the manuscript properly.

We deleted the parts of information and corrected typos error.

10. Show the relationship among serum total protein, albumin, and gamma gap levels, if there have.

We added the analysis of the relationship among serum total protein, albumin, and gamma gap levels and added it in **Methods/**/**Statistical analyses**, **Results** and **Discussion**.

- **Methods/**/**Statistical analyses**, line 235-236; “The relationship among serum total protein, albumin, and gamma gap levels were also tested using correlation coefficients.”
- **Results**, line 252-256; “The correlation coefficients between serum total protein levels, and albumin and gamma gap levels were 0.523 (p<0.001) and 0.777 (p<0.001) in men, and 0.536 (p<0.001) and 0.794 (p<0.001) in women, respectively (data not shown in Table). The correlation coefficients between albumin and gamma gap levels were -0.130 (p<0.001) in men and -0.088 (p<0.001) in women.”
- **Discussion**, line 369-371; “Although levels of albumin and gamma gaps had a weak negative relationship, this study showed an apparent combined effect on the HRs for death between albumin and gamma gap levels.”
